# Multihealth Promotion Programs on Physical Health and Quality of Life in Older Adults: Quasi-Experimental Study

**DOI:** 10.2196/65213

**Published:** 2025-05-01

**Authors:** Li-Yun Lee, Heng-Hsin Tung, George Liao, Su-Ju Liu, Zi-Yu Chen, Yea-Ru Yang

**Affiliations:** 1 Department of Nursing Dayeh University Changhua Taiwan; 2 Department of Nursing National Yang Ming Chiao Tung University Taipei Taiwan; 3 Tungs' Taichung MetroHarbor Hospital Taichung Taiwan; 4 YuSen Biotechnology Co, Ltd Taichung City Taiwan; 5 Keisei Pharmacy Yunlin County Taiwan; 6 YuSen Biotechnology Co, Ltd Taichung Taiwan; 7 Department of Physical Therapy and Assistive Technology National Yang Ming Chiao Tung University Taipei Taiwan

**Keywords:** older adult, body composition, physical activity, health promotion, exercise, nutrition, diet, well-being, quality-of-life, QoL, gerontology, geriatrics

## Abstract

**Background:**

Physical activity and appropriate nutrition are essential for older adults. Improving physical health and quality of life can lead to healthy aging.

**Objective:**

This study aims to investigate the long-term effects of multihealth promotion programs on the physical and mental health of older adults in communities.

**Methods:**

A quasi-experimental method was used to recruit 112 older adults voluntarily from a pharmacy in central Taiwan between April 2021 and February 2023. Participants were divided into an experimental group receiving a multihealth promotion program and a control group with no specific intervention. The study measured frailty, nutritional status, well-being, and quality of life using standardized tools such as the Clinical Frailty Scale (CFS), Mini-Nutritional Assessment-Short Form (MNA-SF), Well-being Scale for Elders, and the EQ-5D-3L. Data were analyzed using descriptive statistics, independent *t* tests, Pearson correlation, and generalized estimating equations.

**Results:**

A total of 112 participants were recruited. There were 64 (57.1%) in the experimental group and 48 (42.9%) in the control group. The experimental group exhibited significantly better quality of life (EQ-5D index) at weeks 12 (*β*=–.59; *P*=.01) and 24 (*β*=–.44; *P*=.04) compared to the control group. The experimental group muscle mass significantly increased at weeks 24 (*β*=4.29; *P*<.01) and 36 (*β*=3.03; *P*=.01). Upper limb strength improved significantly at weeks 12 (*β*=3.4; *P*=.04) and 36 (*β*=5; *P*=.01), while core strength showed significant gains at weeks 12 (*β*=4.43; *P*=.01) and 36 (*β*=6.99; *P*<.01). Lower limb strength increased significantly only at week 12 (*β*=4.15; *P*=.01). Overall physical performance improved significantly at weeks 12 (*β*=5.47; *P*<.01), 24 (*β*=5.17; *P*<.01), and 36 (*β*=8.79; *P*<.01).

**Conclusions:**

The study’s findings highlight the practical benefits of interventions, including physical and social activities and nutritional support, in enhancing the quality of life and general physical health of older adults. This study’s findings have significant implications for clinical practice. These findings can aid in the establishment of effective interventions for older adults.

**Trial Registration:**

ClinicalTrials.gov NCT05412251; https://clinicaltrials.gov/study/NCT05412251

## Introduction

As the challenges of global aging intensify, it is projected that by 2050, individuals aged 60 years and older will account for 22% of the global population [1]. This demographic shift brings a surge in health concerns among older adults, necessitating increased attention. Aging is commonly associated with significant changes in body composition, including substantial loss of muscle strength and mass, which elevates the risk of sarcopenia and functional decline [2]. In addition, older adults are particularly vulnerable to moderate-to-severe malnutrition, further exacerbating health deterioration and reducing quality of life (QoL) [3]. Consequently, health promotion strategies should prioritize improving the nutritional status of older adults and encouraging regular physical activity to support overall health and QoL [4]. Research consistently demonstrates that older adults who engage in regular physical activity experience significantly better physical and mental health outcomes than sedentary individuals, underscoring the undeniable benefits of exercise interventions [5]. As such, exercise interventions are widely regarded as a practical approach to enhancing QoL in older adults [6].

Exercise interventions for older adults encompass a variety of methods, including yoga, breathing exercises, meditation, Baduanjin, Western gymnastics, 24-form Tai Chi, aerobic exercises, outdoor activities, balance, strength, and resistance training [4,5,7]. These interventions typically last 12 weeks, with sessions ranging from 30 to 90 minutes, 2 to 5 times per week, with 3 sessions per week being the most common frequency. However, systematic reviews and meta-analyses reveal that current health promotion studies predominantly focus on single exercise modalities, yielding inconsistent effects on physical function and lacking in-depth exploration of multicomponent exercise interventions, particularly in practical applications and comprehensive outcome assessments [4,5,7]. Specifically, Frost et al [7] conducted a meta-analysis of 7 randomized controlled trials (RCTs), of which only one combined exercise with nutritional interventions. This study found that strength training supplemented with protein significantly improved muscle mass. However, the effects on psychological health and well-being were not adequately explored, highlighting the need for further research on the holistic benefits of multicomponent interventions. Similarly, Iwano et al [8] emphasized in their systematic review that the variation in intervention types (eg, art therapy and social activities) and measurement tools (eg, well-being scales and depression indices) in studies on older adults’ well-being limits the comparability and generalizability of intervention effects, underscoring the necessity for further research. Wei et al [5] advocated for future studies incorporating diverse forms of physical activity to investigate their effects on physical and mental health systematically. They also encouraged older adults to engage in various activities to enhance QoL effectively.

In addition, Sirikul et al [4] conducted a meta-analysis of multicomponent exercise interventions (combining strength, balance, flexibility, and endurance training) and nutritional interventions among frail older adults aged 65 years and above in community settings. Their findings suggest that combining nutritional interventions, such as protein supplementation, with exercise interventions, particularly strength training, effectively improves older adults’ muscle mass and physical function. These findings underscore the potential value of multicomponent strategies and emphasize the importance of integrating exercise and nutritional interventions in future health promotion programs to enhance older adults’ overall health and functional outcomes.

Although exercise interventions are widely recognized for their potential to improve QoL in older adults, their effects on physical function and psychological health remain inconsistent. For example, Sirikul et al [4] conducted a meta-analysis of 21 exercise intervention studies, reporting predominantly positive effects on muscle strength and balance. However, their findings also highlighted the lack of comprehensive evaluations of multicomponent interventions (eg, combining exercise and nutrition). Frost et al [7] reported that only one of 7 RCTs included combined exercise and nutritional interventions, showing that strength training with protein supplementation significantly increased muscle mass. Nevertheless, the effects of this combined intervention on mental and psychological health were not thoroughly explored. Marcos-Pardo PJ et al [9] found that moderate-to-high intensity resistance circuit training significantly improved muscle strength in older adults but had no significant impact on QoL. Conversely, Chittrakul et al [10] reported that multisystem exercise training significantly enhanced QoL in older adults. Existing studies suggest that exercise interventions should be tailored to the physical condition of older adults. Combining social participation, autonomy enhancement, nutritional interventions, physical training, and cognitive activities in multicomponent health promotion programs improves nutritional status and overall physical function. It effectively enhances QoL and well-being while reducing fall risks [4,5,7].

Well-being, a subjective experience characterized by happiness or satisfaction, is a core goal universally pursued by humans. It is closely associated with reduced risks of depression, diminished feelings of hopelessness, improved mental health, enhanced QoL, and strengthened family relationships. For older adults, well-being often stems from life achievements and successful experiences [11]. Enhancing well-being helps older adults maintain health and encourages them to adopt a more positive outlook on life. However, previous research has predominantly focused on treating and managing physical diseases in older adults, with relatively little attention given to well-being [12]. Furthermore, significant disparities remain in the findings of studies investigating the effects of interventions on older adults’ well-being [8]. Studies have shown that social activities significantly improve psychological health by engaging older adults in meaningful, hopeful, and enjoyable interactions [13,14]. Maintaining QoL in older adults is essential for individual health and reflects their adaptability and resilience during aging. High QoL effectively reduces mental health issues, promotes active community participation, strengthens social connections, and alleviates feelings of loneliness [6].

In addition, smart devices provide precise data and enhance older adults’ engagement and adherence to exercise [15,16]. However, further research is needed to compare the effects of specific multicomponent intervention models. This study investigates the comprehensive effects of a multihealth promotion program combining smart exercise devices, social activities, and nutritional prescriptions on the physical function, QoL, and well-being of older adults in community settings. The long-term benefits will also be tracked to provide evidence-based recommendations for promoting healthy aging.

## Methods

### Design and Sample

This study adopted a quasi-experimental design, with participants voluntarily recruited from a pharmacy located in central Taiwan between April 2021 and February 2023. The participants themselves determined allocation to the experimental and control groups. The trial design was registered with ClinicalTrials.gov (identifier: NCT05412251) and followed the CONSORT (Consolidated Standards of Reporting Trials) guidelines (Multimedia Appendix 1). The inclusion criteria for this study were participants (1) aged 65 years or older and (2) fluent in Mandarin (speaking or reading). The study excluded participants who had a history of cognitive impairment, cancer, myocardial infarction, or congestive heart failure. This study used G power software to estimate the sample size. The effect size was calculated based on the pre- and posttest scores of the quality of life of older adults in the intervention group and the control group in a previous study [17]. The effect size was 0.19, the detection power was 0.8, the significance level (α) was .05, and the number of measurements was four times. The results showed that 40 participants were required in each group to ensure that a statistically significant difference in quality of life between the two groups could be detected during the follow-up period. Considering the attrition rate in interventional studies can be as high as 40% [18], at least 56 participants were recruited in each group at baseline. Data were collected at baseline (T0) and 12 weeks, 24 weeks, and 36 weeks postintervention (T1, T2, and T3). The data was collected face-to-face and self-reported by participants.

### Multihealth Promotion Program Intervention

The design of the multihealth promotion program was based on previous studies. We combined 3 intervention strategies: social activities, physical training, and nutritional support, and used artificial intelligence exercise equipment as an assessment tool to measure physical performance during the exercise process [5,13,19]. For 12 weeks, participants in the experimental group engaged in social and physical activities while receiving nutritional support. (1) Social activities: this was combined with activities held at community base C, such as makeup, reading picture books, art classes, board games, and more, for at least 120 minutes, once a week. (2) Physical training: the utilization of artificial intelligence exercise equipment, suitable for middle-aged and older people’s exercise training and approved by the Food and Drug Administration of the Ministry of Health and Welfare. This was carried out at least 60 minutes, 3 times a week, including 8-10 minutes of warm-up exercise before exercise, after exercise, aerobic exercise, muscle strengthening training, and balance training, each requiring 15-20 minutes. Before each exercise, researchers used the exercise assessment and screening for you (EASY) tool to assess participants’ exercise risks, and the AI-enabled exercise equipment will evaluate participants and provide appropriate exercise prescriptions. Throughout the exercise, the researchers observed the physical condition of the participants constantly. If there were signs of discomfort, participants were asked to stop immediately. (3) Nutritional support: nutritionists screened participants’ nutritional status based on the mini nutrition assessment tool and based on personal wishes, provided free protein nutrition products in this plan. During the entire study period, the control group did not receive the intervention program for social activities, physical activity, or nutritional support. They only took measurements and questionnaires at monitoring time points (baseline, 12, 24, and 36 weeks). The on-site nutritionist was available for any questions from the control group.

### Measurements

The study measured demographic and baseline characteristics, frailty, nutrition status, well-being, and quality of life.

### Demographic Data Form

A researcher-made demographic form was prepared based on a literature review. It included age, gender, BMI, education level, lived conditions, and chronic disease.

### Clinical Frailty Scale

The Clinical Frailty Scale (CFS) is used to assess frailty in older adults and was developed by Rockwood et al in 2005. This scale was updated to version 2.0 in 2020. The frailty scale ranges from 1 to 9, where level 1 indicates very fit and level 9 indicates terminally ill, corresponding to a survival time of fewer than 6 months [20].

### Mini-Nutritional Assessment-Short Form

The Mini-Nutritional Assessment-Short Form (MNA-SF) consists of 6 questions that assess key areas of nutritional assessment: decline in food intake, weight loss, mobility, psychological stress or acute disease, neuropsychological problems, and BMI. The total score classifies individuals into 3 categories, normal nutritional status (12-14 points), at-risk of malnutrition (8-11 points), and malnourished (0-7 points). The MNA-SF has been validated in various studies and settings, demonstrating high sensitivity and specificity [21].

### The EASY Tool

The EASY is an exercise screening and assessment tool designed to help older adults choose safe and effective exercise modalities and physical activity programs. There are 6 questions, each of which can be answered with “yes” or “no.” If the answer to one of the questions is “yes,” the participant must find a health care provider to discuss an appropriate exercise plan before exercising [22].

### Well-Being Scale for Elders

The Well-being Scale for Elders is a 9-item self-report questionnaire, including emotional, psychological, and social well-being. The higher scores indicate better well-being. It has good reliability and construct validity and can be effectively used to measure the happiness of older adults. The Cronbach α is 0.91, and the factor analysis results explain 59.67% of the variation [12]. In this study, Cronbach α is 0.82.

### EQ-5D-3L Instrument

The EQ-5D 3L is a standardized instrument developed by the EuroQol Group to measure general health status. It is widely used in both clinical and economic evaluations to assess health-related quality of life. The EQ-5D index comprises 5 dimensions: mobility, self-care, daily activities, pain or discomfort, and anxiety or depression. Each dimension has 3 levels of severity (no problems, moderate problems, and extreme problems). Higher scores on this index indicate a poorer health-related quality of life. EQ-VAS (European Quality of Life Visual Analogue Scale) is a visual analog scale ranging from 0 to 100, with higher scores indicating a better-perceived quality of life. Respondents evaluate their overall health on a scale from 0, indicating the worst possible health, to 100, indicating the best possible health [23]. The EQ-5D-3L has demonstrated good reliability, validity, and sensitivity for measuring health-related quality of life [24]. In this study, Cronbach α is 0.8.

### Statistical Analysis

All data were analyzed using the SPSS software program (version 24; IBM Corp). This research uses descriptive statistics to analyze frequencies, means, and SD. Differences in outcome variables at baseline between the two groups are analyzed using the chi-square test and an independent *t* test. The Generalized Estimating Equations (GEE) analyze changes over time, group differences, and group-by-time interactions, including body composition, frailty, well-being, and quality of life.

### Ethical Considerations

This study was conducted with the utmost ethical considerations and approved by the National University Institutional Review Board (YM110168EF). All participants were respected, and their autonomy was upheld, as they provided written informed consent before enrollment. Participants were fully informed by a research assistant about the study’s purpose, procedure, benefits, and potential risks, ensuring transparency and respect for their rights and welfare. The participant data has been deidentified for privacy concerns.

## Results

**Figure 1:**
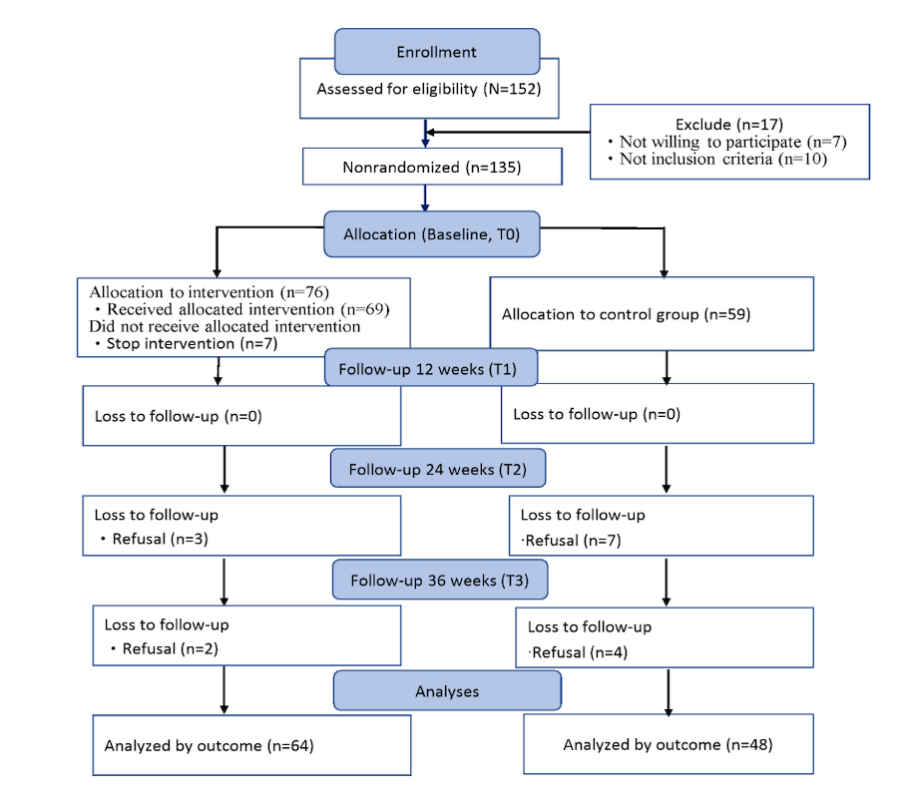
Flowchart of participants’ recruitment.

In this study, after excluding ineligible participants and those who dropped out, a total of 112 older adults (64 in the experimental group and 48 in the control group) completed the study, with 15.8% and 18.6% attrition rates, respectively (Figure 1). Most participants were female (67%), with an average BMI of 24.5 (3.6) kg/m². Overall, 23.2% (26/112) of the participants lived alone; the average frailty score was 2.2 (SD 0.9). Baseline characteristics showed good homogeneity between the 2 groups. As of February 2023, we have recruited 152 participants (Table 1).

Table 2 reveals average baseline BMI in the control group as 24.13 (SD 3.89) kg/m², which slightly decreased to 24.06 (SD 3.63) kg/m² at week 36. Frailty scores rose from 2.31 to 2.45, while the average well-being scores improved from 38.21 (SD 5.42) to 38.59 (SD 5.45). Quality-of-life scores showed improvement, decreasing from an average of 5.50 (SD 1.07) to 5.35 (SD 0.90). In the experimental group, average baseline BMI was 24.79 (SD 3.34) kg/m², which reduced to 24.43 (SD 3.42) kg/m² by week 36. Frailty scores increased slightly from 2.11 to 2.16, and average well-being scores rose from 37.86 (SD 5.99) to 38.91 (SD 4.97). Quality-of-life scores improved significantly, decreasing from an average of 5.98 (SD 1.46) to 5.42 (SD 0.79).

Table 3 shows that the GEE analysis revealed significant BMI reductions across all participants in the first 12 weeks. At week 36, the experimental group had a significantly lower BMI compared to the control group. Notably, BMI reductions were more pronounced in the experimental group at weeks 12 (*β*=–.22; *P*=.02) and 24 (*β*=–.20; *P*=.06), with a smaller decrease at week 36 (*β*=.15; *P*=.23). The interaction effect between groups at week 36 was significant (*β*=–.51; *P*<.001), highlighting a greater BMI reduction in the experimental group. Frailty assessment showed no significant interaction between time and group, suggesting stable frailty levels during the intervention. Similarly, no significant differences in well-being were observed. However, the experimental group exhibited significantly better quality of life (EQ-5D index) at weeks 12 (*β*=–.59; *P*=.01) and 24 (*β*=–.44; *P*=.04) compared to the control group. No significant differences were observed in EQ-VAS scores between groups.

**Table 1:** Baseline characteristics (N=112).

Variables		Total (N=112)	EG^a^ (n=64)	CG^b^ (n=48)	Values (*χ*^2^/*t*)		*P* value	
Age (year), mean (SD)^a^		75.1 (6.2)	75.81 (5.1)	74.16 (1.1)	–1.32		.19^c^	
**Sex, n (%)**						4.36		.04^d^
	Female	75 (67)	48 (75)	27 (56.3)				
	Male	37 (33)	16 (25)	21 (43.8)				
BMI (kg/m^2^), mean (SD)		24.5 (3.6)	24.8 (3.3)	24.1 (3.9)	–0.96		.34^c^	
**Education level, n (%)**						4.82		.19^d^
	None	13 (11.6)	7 (10.9)	6 (12.5)				
	Elementary	56 (50)	36 (56.3)	20 (41.7)				
	Junior high	10 (8.9)	7 (10.9)	3 (6.3)				
	Senior high and above	33 (29.5)	14 (21.9)	19 (39.6)				
**Lived conditions, n (%)**						1.19		.28^d^
	Alone	26 (23.2)	13 (20.3)	6 (12.5)				
	With family	86 (76.8)	51 (79.7)	42 (87.5)				
**Chronic disease, n (%)**						3.04		.08^d^
	No	86 (76.8)	53 (82.8)	33 (68.8)				
	Yes	26 (23.2)	11 (17.2)	15 (31.3)				
Frailty^e^, mean (SD)		2.2 (0.9)	2.1 (0.9)	2.3 (0.9)	1.18		.24^c^	
Well-being^f^, mean (SD)		38 (5.7)	37.7 (6)	38.2 (5.4)	0.32		.75^c^	
EQ-5D index, mean (SD)		5.7 (1.3)	5.9 (1.4)	5.5 (1.1)	–1.72		.09^c^	
EQ-VAS^g^, mean (SD)		80.6 (9.7)	81.2 (8.2)	79.7 (11.3)	–0.84		.4^c^	

^a^EG: experimental group.

^b^CG: control group.

^c^Conducted using independent *t* test (*df*=110).

^d^Conducted using χ^2^ test (*df*=1,3).

^e^Frailty: The Clinical Frailty Scale.

^f^Well-being: Well-being scale for Elders.

^g^EQ-VAS: European Quality of Life Visual Analogue Scale.

**Table 2:** Changes at different periods between the experimental group and the control group (N=112).

Variable and group			Baseline, mean (SD)		12 Weeks, mean (SD)		24 Weeks, mean (SD)		36 Weeks, mean (SD)
**BMI (kg/m^2^)**									
	EG^a^	24.79 (3.34)		24.49 (3.46)		24.39 (3.40)		24.43 (3.42)	
	CG^b^	24.13 (3.89)		23.91 (3.77)		23.93 (3.73)		24.06 (3.63)	
**Frailty^c^**									
	EG	2.11 (0.88)		2.06 (0.81)		2.12 (0.85)		2.16 (0.84)	
	CG	2.31 (0.93)		2.46 (0.80)		2.46 (1.01)		2.45 (1.02)	
**Well-being^d^**									
	EG	37.86 (5.99)		37.53 (4.92)		38.92 (4.92)		38.91 (4.97)	
	CG	38.21 (5.42)		37.65 (5.70)		38.33 (4.82)		38.59 (5.45)	
**EQ–5D index**									
	EG	5.98 (1.46)		5.50 (0.81)		5.42 (0.85)		5.42 (0.79)	
	CG	5.50 (1.07)		5.60 (1.14)		5.38 (0.96)		5.35 (0.90)	
**EQ-VAS^e^**									
	EG	81.23 (8.24)		80.63 (8.30)		82.42 (10.11)		83.20 (9.16)	
	CG	79.69 (11.30)		80.35 (10.68)		81.33 (10.14)		81.74 (10.04)	

^a^EG: experimental group.

^b^CG: control group.

^c^Frailty: the Clinical Frailty Scale.

^d^Well-being: Well-being scale for Elders.

^e^EQ-VAS: European Quality of Life Visual Analogue Scale.

**Table 3:** Generalized estimating equations analysis of BMI, frailty, happiness, and quality of life in older participants (N=112).

Parameter				*β*			SE				95% Wald CI		*P* value		
**BMI (kg/m^2^)**															
		(Intercept)	24.13			0.56				23.04 to 25.22		<.001			
		EG^a^	0.66			0.69				–0.69 to 2.02		.34			
		CG^b^ (reference group)	0^a^			—^c^				—		—			
		36 weeks	0.15			0.12				–0.10 to 0.39		.23			
		24 weeks	–0.20			0.10				–0.40 to 0.01		.06			
		12 weeks	–0.22			0.09				–0.41 to –0.03		.02			
		Baseline	0^a^			—				—		—			
		EG*36 weeks	–0.51			0.15				–0.81 to –0.21		.001			
		EG*24 weeks	–0.21			0.14				–0.48 to 0.07		.14			
		EG*12 weeks	–0.08			0.13				–0.33 to 0.17		.53			
		EG* baseline	0^a^			—				—		—			
**Frailty^d^**															
		(Intercept)	2.31			0.13				2.05 to 2.57		.001			
		EG	–0.20			0.17				–0.54 to 0.13		.24			
		CG (reference group)	0^a^			—				—		—			
		36 weeks	0.15			0.09				–0.04 to 0.33		.13			
		24 weeks	0.15			0.09				–0.03 to 0.32		.1			
		12 weeks	0.15			0.10				–0.05 to 0.34		.14			
		Baseline	0^a^			—				—		—			
		EG*36 weeks	–0.10			0.12				–0.34 to 0.15		.43			
		EG*24 weeks	–0.07			0.11				–0.29 to 0.15		.54			
		EG*12 weeks	–0.19			0.12				–0.43 to 0.04		.11			
		EG* baseline	0^a^			—				—		—			
**Well-being^e^**															
		(Intercept)	38.21			0.77				36.69 to 39.73		<.001			
		EG	–0.35			1.07				–2.45 to 1.76		.75			
		CG (reference group)	0^a^			—				—		—			
		36 weeks	0.30			0.81				–1.28 to 1.88		.71			
		24 weeks	0.13			0.87				–1.58 to 1.83		.89			
		12 weeks	–0.56			0.82				–2.18 to 1.05		.49			
		Baseline	0^a^			—				—		—			
		EG*36 weeks	0.75			1.12				–1.44 to 2.94		.50			
		EG*24 weeks	0.94			1.20				–1.41 to 3.28		.43			
		EG*12 weeks	0.23			1.03				–1.79 to 2.26		.82			
		EG* baseline	0^a^			—				—		—			
**EQ-5D index**															
		(Intercept)	5.55			0.15				5.2 to 5.8		.001			
		EG	0.48			0.24				0.02 to 0.95		.04			
		CG (reference group)	0^a^			—				—		—			
		36 weeks	–0.16			0.16				–0.47 to 0.14		.30			
		24 weeks	–0.13			0.15				–0.42 to 0.17		.41			
		12 weeks	0.10			0.11				–0.11 to 0.32		.35			
		Baseline	0^a^			—				—		—			
		EG*36 weeks	–0.40			0.23				–0.84 to 0.04		.08			
		EG*24 weeks	–0.44			0.21				–0.85 to –0.03		.04			
		EG*12 weeks	–0.59			0.19				–0.96 to –0.21		.01			
		EG* baseline	0^a^			—				—		—			
**EQ-VAS^f^**															
	(Intercept)		79.69			1.61				76.52 to 82.85		.01			
	EG		1.55			1.91				–2.19 to 5.29		.42			
	CG (reference group)		0^a^			—				—		—			
	36 weeks		1.74			1.46				–1.11 to 4.6		.23			
	24 weeks		1.64			1.04				–0.39 to 3.68		.11			
	12 weeks		0.66			0.71				–0.73 to 2.06		.35			
	Baseline		0^a^			—				—		—			
	EG*36 weeks		0.22			1.96				–3.62 to 4.07		.91			
	EG*24 weeks		–0.46			1.81				–4 to 3.08		.80			
	EG*12 weeks		–1.28			1.23				–3.68 to 1.13		.30			
		EG* baseline			0^a^			—	—					—	

^a^EG: experimental group.

^b^CG: control group.

^c^Not applicable.

^d^Frailty: the Clinical Frailty Scale.

^e^Well-being: Well-being scale for Elders.

^f^EQ-VAS: European Quality of Life Visual Analogue Scale.

Table 4 shows that GEE analysis revealed no significant changes in body fat percentage at weeks 12 (*β*=–.03; *P*=.69) and 36 (*β*=–.31; *P*=.27). However, muscle mass significantly increased at weeks 24 (*β*=4.29; *P*<.001) and 36 (*β*=3.03; *P*=.01), demonstrating the intervention’s effectiveness in enhancing muscle mass. Upper limb strength improved significantly at weeks 12 (*β*=3.40; *P*=.04) and 36 (*β*=5; *P*=.01), while core strength showed significant gains at weeks 12 (*β*=4.43; *P*=.01) and 36 (*β*=6.99; *P*<.001). Lower limb strength increased significantly only at week 12 (*β*=4.15; *P*=.01). Overall physical performance improved significantly at weeks 12 (*β*=5.47; *P*<.001), 24 (*β*=5.17; *P*<.001), and 36 (*β*=8.79; *P*<.001).

**Table 4:** Body composition in the experimental group analyzed by generalized estimating equations (n=64).

Outcome			B		SE		95% CI		Wald χ^2^		*P*
**Body fat percentage**											
	Intercept	3.77		0.27		3.24 to 4.29		196.56		<.001	
	36 weeks^a^	–0.31		0.28		–0.86 to 0.24		1.24		.27	
	24 weeks^a^	–0.42		0.28		–0.97 to 0.13		2.27		.13	
	12 weeks^a^	–0.03		0.08		–0.19 to 0.13		0.15		.69	
**Muscle content**											
	Intercept	33.06		1.2		30.7 to 35.42		753.74		<.001	
	36 weeks^a^	3.03		1.24		0.61 to 5.47		6		.01	
	24 weeks^a^	4.29		1.21		1.92 to 6.66		12.58		<.001	
	12 weeks^a^	–0.67		0.73		–2.10 to 0.77		0.83		.36	
**Upper limb score**											
	Intercept	65.91		1.36		63.25 to 68.57		2363.36		<.001	
	36 weeks^a^	5		1.98		1.13 to 8.86		6.43		.01	
	24 weeks^a^	2.42		1.93		–1.37 to 6.20		1.57		.21	
	12 weeks^a^	3.4		1.67		0.12 to 6.68		4.13		.04	
**Core score**											
	Intercept	65.05		1.1		62.9 to 67.2		3520.3		<.001	
	36 weeks^a^	6.99		2		3.07 to 10.91		12.2		<.001	
	24 weeks^a^	1.02		1.80		–2.5 to 4.54		0.33		.57	
	12 weeks^a^	4.43		1.44		1.6 to 7.25		9.45		.01	
**Lower limb score**											
	Intercept	67.48		1.35		64.84 to 70.12		2508.61		<.001	
	36 weeks^a^	3.23		2		–0.7 to 7.15		2.6		.11	
	24 weeks^a^	0.91		1.96		–2.94 to 4.76		0.22		.64	
	12 weeks^a^	4.15		1.4		1.42 to 6.88		8.85		.01	
**Comprehensive sports score**											
	Intercept	62.38		1.05		60.32 to 64.43		3553.19		<.001	
	36 weeks^a^	8.79		1.36		6.12 to 11.46		41.58		<.001	
	24 weeks^a^	5.17		1.21		2.79 to 7.55		18.1		<.001	
	12 weeks^a^	5.47		1.37		2.78 to 8.16		15.83		<.001	

^a^Reference group is the baseline.

## Discussion

### Principal Findings

This study’s findings indicate that an intervention program encompassing exercise, social activities, and nutritional prescriptions significantly improved muscle mass, muscle strength, and QoL in the experimental group. Exercise programs have been demonstrated to enhance physical function, body composition, QoL, and muscle strength in older adults [6,9,10,16]. However, their effects on mental health and subjective QoL are less consistent and warrant further investigation. In this study, the experimental group’s QoL (measured by the EQ-5D index) was significantly higher than the control group at weeks 12 and 24. However, no significant difference was observed in EQ-VAS scores. By week 36, the experimental group showed a trend of improvement in QoL, suggesting that the intervention may have long-term benefits for mental health. These findings align with Marcos-Pardo et al [6,9], who reported that resistance training interventions improved lean body mass, muscle strength, and QoL. In a randomized controlled trial (RCT) by P J Marcos-Pardo et al [6], the experimental group participated in an 8-week outdoor resistance training program involving bi-weekly sessions with weight machines. Results revealed significant increases in lean body mass, reductions in fat mass, and improvements in muscle strength (arm and leg), functional activity, and QoL. At the same time, the control group experienced declines in muscle strength, activity capacity, dynamic balance, and QoL. Another RCT by Marcos-Pardo et al [9] demonstrated that older adults in the experimental group engaging in moderate-to-high intensity resistance circuit training for 12 weeks experienced significant reductions in body weight, fat mass, and BMI, along with significant improvements in lean body mass, functional independence, and upper and lower limb strength. However, no significant changes in body fat or visceral fat were observed in the experimental group during the study period. Chittrakul et al [10] conducted an RCT involving prefrail older adults with a 12-week exercise intervention and a two-year follow-up. The experimental group exhibited significant improvements in muscle strength at week 12, with enhancements in knee extension, balance, and reaction time persisting through week 24. The experimental group’s health-related QoL was significantly higher than the control group at week 12. However, by week 24, despite the control group engaging in thrice-weekly stretching exercises, there was no significant difference in QoL between the groups, contrasting with this study’s sustained improvements in the experimental group beyond week 24. This study also observed a BMI reduction at week 36, differing from previous findings [9], underscoring the need to explore further BMI changes and their relationships with muscle mass and strength.

Unlike the QoL findings, this study did not detect significant differences in well-being between the experimental and control groups, contrasting with the findings of Lai et al [25] and Jung et al [26]. The quasi-experimental study by Lai et al [25] demonstrated that a 6-day outdoor activity program, including kayaking, rafting, hiking, and camping, significantly improved well-being, self-image, and mental health among older adults, differing from this study’s results. Similarly, Jung et al [26] reported that a 6-week integrated cognitive function improvement program significantly enhanced cognitive function and well-being in the experimental group, with no significant changes observed in the control group. The lack of significant well-being improvements in this study might be attributable to participant characteristics, cultural contexts, or the predominantly indoor nature of the intervention activities. Notably, previous research [27] has highlighted the mediating and moderating roles of outdoor and group exercise in enhancing well-being, emphasizing the psychological benefits of these activities. This underscores the importance of environmental and social factors in promoting well-being. The findings related to well-being suggest that additional intervention components beyond physical and nutritional strategies may be necessary. Factors such as depression risk, social relationships, and leisure activities have been shown to influence well-being [11]. Furthermore, Yang et al [27] emphasized that outdoor environments and group interactions significantly enhance well-being by fostering social connections and providing meaningful engagement. Future studies should consider integrating these elements to maximize the psychological benefits of multicomponent interventions.

Exercise and nutritional interventions have positively affected older adults’ physical function and weight management. Liao et al [19] showed that combining exercise with nutritional support improved grip strength and walking speed despite limited changes in lean body mass. Similarly, in this study, while there were no significant changes in body fat or visceral fat in the experimental group, muscle mass significantly increased at week 24, with effects sustained through week 36. Upper limb strength improved significantly up to week 36, whereas lower limb strength showed significant improvements only at week 12. These findings highlight the need for more targeted designs in future interventions to address lower limb training.

This study underscores the potential of multidisciplinary active aging programs to improve physical function, QoL, and muscle strength among community-dwelling older adults. Such programs have been shown to reduce frailty risk, enhance flexibility, and promote social participation [28]. However, single-component nutritional interventions may have limited effects on individuals without malnutrition or unintentional weight loss [4], consistent with this study’s findings. Clinicians should prioritize integrating physical, nutritional, and social interventions into comprehensive strategies tailored to older adults’ needs. Muscle mass and muscle strength are positively correlated, and increasing muscle mass is key to improving muscle strength in older adults. Muscle mass and body fat percentage are important predictors of muscle strength. With aging, physical fitness declines, and body composition changes (eg, decreased muscle mass and increased obesity) are common [29]. Enhancing muscle strength is critical for clinical health care providers, contributing to improved stability, coordination, flexibility, and balance, ultimately reducing injury risk [30]. As global life expectancy rises and the proportion of older adults increases, promoting healthy aging is not merely a slogan but an urgent priority. However, physical inactivity and nutritional issues are prevalent among older adults [31], and these challenges are closely tied to their health and QoL [3]. While this study did not delve deeply into the influencing factors of QoL, well-being, and nutritional status, the results suggest these areas are important directions for future research.

### Limitations

This study has several limitations that must be noted. First, the quasi-experimental design may introduce selection bias, as participants were not randomized. Second, the predominantly indoor nature of the activities may have limited the potential psychological benefits. Third, this study did not explore the influence of individual variability in adherence and baseline activity levels on the outcomes. Fourth, it was unable to determine the potential for individuals in either the experimental or control group to participate in additional outdoor or physical activities outside of the intervention program, which could have influenced the results. Further studies should address these limitations by employing randomized controlled trial designs, which include outdoor and group-based activities, examining the effects of participant adherence and baseline characteristics, and tracking any additional activities performed outside of the intervention.

### Conclusion

In conclusion, this study confirms the beneficial effects of multicomponent intervention programs on older individuals’ physical function and quality of life. However, the limited benefits on well-being underline the importance of more comprehensive solutions that consider psychological and social aspects. Future studies should concentrate on the long-term assessment of nutritional status, quality of life, and well-being and the creation of individualized intervention programs that incorporate outdoor activities to promote active and healthy aging.

## Appendix

Multimedia Appendix 1 CONSORT 2010 checklist.

## Abbreviations

CFS: Clinical Frailty Scale
CONSORT: Consolidated Standards of Reporting Trials
EASY: exercise assessment and screening for you
EQ-VAS: European Quality of Life Visual Analogue Scale
GEE: Generalized Estimating Equations
MNA-SF: Mini-Nutritional Assessment-Short Form
QoL: quality of life
RCT: randomized clinical trial

## Data Availability

The datasets generated or analyzed during this study are available from the corresponding author on reasonable request.
